# Long-Term *In Vitro* Degradation of a High-Strength Brushite Cement in Water, PBS, and Serum Solution

**DOI:** 10.1155/2015/575079

**Published:** 2015-10-26

**Authors:** Ingrid Ajaxon, Caroline Öhman, Cecilia Persson

**Affiliations:** Division of Applied Materials Science, Department of Engineering Sciences, Uppsala University, Sweden

## Abstract

Bone loss and fractures may call for the use of bone substituting materials, such as calcium phosphate cements (CPCs). CPCs can be degradable, and, to determine their limitations in terms of applications, their mechanical as well as chemical properties need to be evaluated over longer periods of time, under physiological conditions. However, there is lack of data on how the* in vitro* degradation affects high-strength brushite CPCs over longer periods of time, that is, longer than it takes for a bone fracture to heal. This study aimed at evaluating the long-term* in vitro* degradation properties of a high-strength brushite CPC in three different solutions: water, phosphate buffered saline, and a serum solution. Microcomputed tomography was used to evaluate the degradation nondestructively, complemented with gravimetric analysis. The compressive strength, chemical composition, and microstructure were also evaluated. Major changes from 10 weeks onwards were seen, in terms of formation of a porous outer layer of octacalcium phosphate on the specimens with a concomitant change in phase composition, increased porosity, decrease in object volume, and mechanical properties. This study illustrates the importance of long-term evaluation of similar cement compositions to be able to predict the material's physical changes over a relevant time frame.

## 1. Introduction

Bone loss and fractures, due to, for example, cancer or osteoporosis, may call for the use of bone substituting materials. When bone regrowth is desired, resorbability is needed from the bone repair material, where the ideal degradation rate would be the same as that of the osseous tissue formation [1]. Calcium phosphate cements (CPCs) have the potential to be used as such a bone repair material, as they can be designed to slowly resorb over time and they allow bone ingrowth and can stimulate bone formation [2]. In fact, there are already several bone replacement products on the market based on calcium phosphates [3]. However, since CPCs are inherently brittle and therefore commonly not used alone, nor are they approved for use alone, in load-bearing applications, their mechanical as well as chemical properties need to be evaluated over longer periods of time under physiological conditions, to be able to determine when these cements could be used and what their limitations are.

Depending on the type of CPC used, the materials have different degradation rates* in vitro*: the cement can either be stable or degrade through dissolution, by releasing calcium and phosphate ions; disintegration, by fragmentation; and/or conversion to a thermodynamically more stable phase [2, 4]. Upon* in vitro* incubation in foetal bovine serum (FBS) and a phosphate buffered saline (PBS) solution containing calcium ions for 4 weeks, Grover et al. found that brushite cements exhibited a major loss in mass (almost 70% in FBS), an increasing amount of hydroxyapatite (HA), and considerable fragmentation of the cements [5]. However, the cements studied had a remarkably low initial compressive strength (1 ± 0.2 MPa), likely due to the large amount of unreacted beta-tricalcium phosphate (*β*-TCP; cements consisted of 66 wt%  *β*-TCP and 34 wt% brushite) and high liquid-to-powder (L/P) ratio (0.6 mL/g), therefore possibly having a limited use clinically. In a subsequent degradation study by Grover et al., the evolution of strength and porosity of a brushite cement was studied and it was found that the strength decreased almost by half (from 14 ± 2 to 8 ± 3 MPa) over a time period of 4 weeks, with a concomitant porosity increase (from 21 ± 1 to 36 ± 1%). However, in this study, no HA formation could be seen in neither PBS (exact content not stated) nor FBS up until the study was terminated after almost 13 weeks of incubation, likely due to the presence of pyrophosphate ions inhibiting the precipitation of HA [6]. de Oliveira Renó et al. showed that ageing brushite cements in Ringers solution for up to 4 weeks also lead to an increase in porosity (not quantified) and a decrease in strength (from 5.3 ± 0.9 to 1.8 ± N/A MPa) and concluded, since no crystalline phases other than brushite and *β*-TCP were present, that the degradation took place by dissolution of those two phases [7]. Alge et al. found that dissolution was the key degradation mechanism in a monocalcium phosphate monohydrate (MCPM)/*β*-TCP-based brushite cement, contrary to a MCPM/HA-based brushite cement which underwent conversion of brushite to HA, when specimens were soaked in PBS for up to 2 weeks [8]. After 4 weeks of* in vitro* incubation of brushite cements in distilled water and simulated body fluid (SBF, content not stated), Cama et al. showed conversion of some of the brushite to small amounts of monetite, with the presence of octacalcium phosphate (OCP) and increasing amounts of monetite when aged in distilled water [9]. Bohner et al. showed that the composition of brushite cements was stable over time when incubated for just over 2 weeks in deionized water under physiological temperature, whereas monetite was formed when the temperature was increased [10]. The above mentioned studies investigated the* in vitro* degradation behaviour of low-strength brushite cements, that is, having strengths lower than 15 MPa (24 hours after setting), with Cama et al. [9] being one exception (27.6 ± 3.3 MPa). Neither Alge et al. [8] nor Bohner et al. [10] evaluated the strength of the cements.

Brushite cements with strengths of 74.4 ± 10.7 MPa have recently been developed [11], which may have an increased potential for use in load-bearing applications where the load is mainly compressive, for example, certain types of vertebral compression fractures [12]. To the best of the authors' knowledge, no investigation of how* in vitro* degradation affects the mechanical properties, porosity, and pore size distribution including both macro- and microstructure of high-strength brushite cements has been reported in the literature. The degradation of these cements may be very different to that of lower strength cements, in particular since the porosity is generally much lower [13]. Finally, most previous degradation studies have been terminated after 2–4 weeks of incubation time [5–10, 14, 15], even though bone fracture healing takes considerably longer than that (ranging from ~6 weeks up to several months or longer, depending on fracture site, age, and health status of the injured patient) [16, 17]. Three longer degradation studies have been undertaken (almost 13 weeks [6], 16 weeks [18], and 1 year [19]). However, in the study by Grover et al. [6], neither porosity nor compressive strength was evaluated after 4 weeks of incubation time, in the study by Rousseau and Lemaître [18] no quantitative data was presented, and in the study by Tan et al. [19] the endpoint of porosity evaluation and phase composition was after 14 days of* in vitro* incubation. Hence, there is a need to evaluate the degradation properties of brushite cements, in terms of, for example, mechanical strength, porosity, pore size distribution, and phase composition over longer periods of time, that is, longer than it takes for a bone fracture to heal, especially for high-strength brushite cements. Such data is of uttermost importance for predictions of when brushite cements have the potential to be used clinically, not only for an estimation of mechanical properties over time, but also for the possible biological response, which also depends on the porosity and its size distribution [2]. Knowledge of the porosity and mechanical properties of these cements over time is also important for the development and validation of computational models that include these types of materials.

As a complement to previously used methods, microcomputed tomography (micro-CT) is a method that can be used to study material degradation behaviour. It is a versatile visualization technique that can be utilised to image internal structures of objects in the micrometer range. Its nondestructive nature could make it ideal for longitudinal evaluation of* in vitro* degradation properties as well as for forming the base for and providing validation data to computational models.

This study aimed to evaluate the long-term degradation behaviour of a high-strength brushite cement. Micro-CT was used to evaluate the degradation nondestructively, which has, to the best of the authors' knowledge, never been done before. By using micro-CT, the degradation propagation in 3D as well as the macroporosity of the cement could be studied. Due to its limited resolution, the evaluation was complemented with gravimetric analysis, using a solvent exchange method [13]. Compositional and microstructural analysis was also performed, using X-ray diffraction (XRD) and scanning electron microscopy (SEM). In order to evaluate how the chemical surroundings of the cement affected its properties, the degradation of the cement was studied in three different liquids.

## 2. Materials and Methods

### 2.1. Cement Preparation

A high-strength CPC made of brushite was the focus of this study. The same cement composition has been thoroughly studied before, in terms of initial porosity and mechanical properties [11, 13]. To prepare the cement paste, MCPM (Scharlau, Sentmenat, Spain) was first sieved to obtain particle sizes below 75 *μ*m. Sieved MCPM was then mixed with *β*-TCP (Sigma-Aldrich, St. Louis, MO, USA) in a 45 : 55 molar ratio, together with 1 wt% disodium dihydrogen pyrophosphate (SPP; Sigma-Aldrich, St. Louis, MO, USA) acting as a retardant [20]. Citric acid (0.5 M, aq.) was used as the liquid phase and was thoroughly mixed with the powder phase (MCPM, *β*-TCP, and SPP) at an L/P ratio of 0.22 mL/g in a mechanical mixing device (Cap-Vibrator, Ivoclar Vivadent AG, Schaan, Liechtenstein) for 1 min. Cylindrical specimens, 6 mm in diameter, were moulded and left to set for 5 min at room temperature (RT, 21 ± 1°C), before being immersed into 40 mL of PBS (Sigma-Aldrich, St. Louis, MO, USA; containing 0.01 M phosphate buffer, 0.0027 M potassium chloride, and 0.137 M sodium chloride, pH 7.4) and kept at 37°C for 24 hours. After 24 hours, the set specimens were wet-polished plane-parallel using SiC paper, to a final height of 12 mm (sample dimensions according to ASTM F 451-08 [21]).

### 2.2. *In Vitro* Degradation

Three different liquids were used for the* in vitro* degradation experiments: double distilled H_2_O, PBS (same composition as above), and a serum solution. All nonsterilized liquids were sterilized (sterile filtered) prior to use. The serum solution was prepared by diluting 10% fetal bovine serum (HyClone, Thermo Scientific, Cramlington, UK) in PBS (same composition as above), with addition of 0.1 wt% sodium azide, as bactericidal agent, a solution similar to what has previously been used in degradation studies of brushite cements [5, 6], however herein diluted in accordance with the recommendations specified in ASTM F 732-00 [22]. These three liquids were selected to simulate* in vivo* conditions (PBS, serum solution) but also to investigate whether the* in vitro* testing can be simplified (H_2_O). Cement specimens were sterilized in water under UV light (having a peak wavelength of 254 nm, 45 min per side) prior to being immersed in the liquid, 20 mL per specimen (corresponding to a liquid-cement-volume ratio of 60 [5]), and kept at 37°C until testing in allocated experiments. All liquids were refreshed once per week. Measurements of the pH value of all liquids initially and after 5, 10, 15, 20, and 25 weeks (before refreshing) showed that it was approximately neutral and stable throughout the experiment (pH 6.6–7.5). All experiments were performed under sterile conditions.

**Figure 1:**
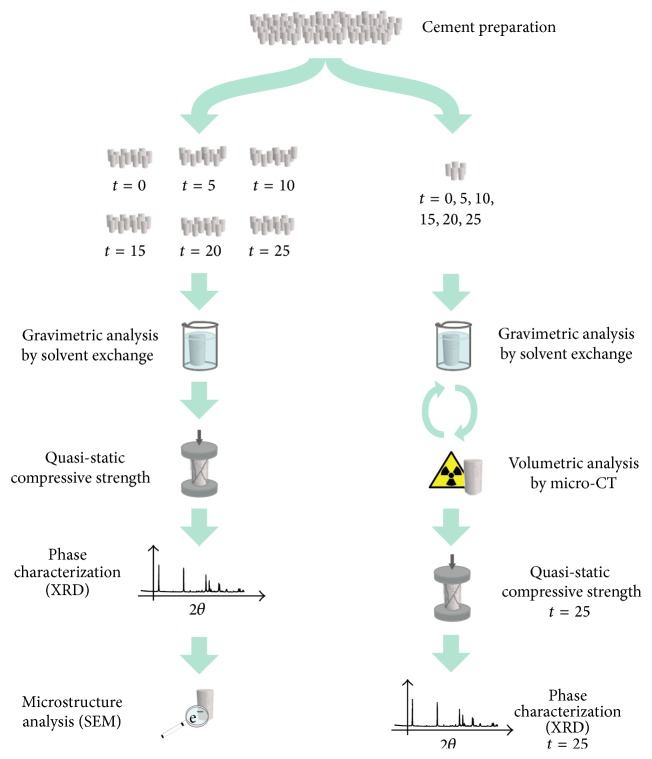
Schematic of the experimental setup. The experiments were repeated for specimens kept in three different degradation liquids: H_2_O, PBS, and serum solution.

Two series of experiments were carried out, one where the degradation was studied with micro-CT and one where the degradation was studied in terms of mechanical properties, phase composition using XRD, and SEM. The wet porosity of the specimens was evaluated by solvent exchange in both series [13]. A schematic of the experiments is shown in Figure 1. Experiments were performed after the cements had set for 24 hours in PBS at 37°C (hereafter referred to as time point 0) and after the cements had been kept in liquid for 5, 10, 15, 20, and 25 weeks, if not otherwise specified. For the micro-CT study, the same cement specimens were analysed at every time point.

### 2.3. Gravimetric Analysis by Solvent Exchange

Solvent exchange has been thoroughly investigated and validated as a wet porosity measurement method for brushite cements in a previous study and more details about the method can be found in [13], but it is briefly summarized as follows: At every time point, the apparent volume, *V*_*a*_, of each specimen was determined using a density kit (Mettler Toledo, Greifensee, Switzerland) based on Archimedes' principle. *V*_*a*_ was calculated using

(1) $$\begin{array}{c} V_{a}=\frac{m_{air}-m_{H_{2}O}}{\rho_{H_{2}O}}, \end{array}$$

where *m*_air_ is the mass of the wet specimen in air, *m*_H_2_O_ is the mass of the wet specimen in water, and *ρ*_H_2_O_ is the density of water (approximately 1 g/cm^3^ at RT). Each specimen was then immersed into 10 mL of isopropanol (VWR, Fontenay-sous-Bois, France) and kept at RT for approximately 24 hours. The mass of each specimen was recorded and the total open porosity (i.e., pores that can be penetrated by the isopropanol molecule), Φ, of the specimen was calculated using

(2) $$\begin{array}{c} \Phi \left(\;\%\;\right)=\frac{\left(m_{air}-m_{solvent}\right)/\left(\rho_{H_{2}O}-\rho_{solvent}\right)}{V_{a}}\times 100, \end{array}$$

where *m*_solvent_ is the mass of the specimen after 24 hours in isopropanol (complete solvent exchange) and *ρ*_solvent_ is the density of isopropanol (0.786 g/cm^3^ at RT).

### 2.4. Volumetric Analysis by Micro-CT

Cement specimens (5 replicates per liquid) were analysed by micro-CT (SkyScan 1172, Bruker microCT, Kontich, Belgium) by placing them on top of each other with the long axis oriented vertically in a poly(methyl methacrylate) container filled with double distilled H_2_O. The scanner operated at a source voltage of 100 kV and a current of 100 *μ*A and using a Cu-Al filter. Images were acquired using an isotropic pixel size of 6.9 *μ*m^2^. After micro-CT scanning, specimens were sterilized (as described above) prior to being immersed in fresh liquid and kept at 37°C until the next time point. All liquids were refreshed once per week. The micro-CT images were reconstructed using NRecon (Bruker microCT, Kontich, Belgium). The images were binarized to separate the specimen from the background, using a global thresholding procedure. Thresholds were visually determined for all acquired datasets at the first time point and thereafter the average value was applied to all specimens at all time points. Calculations of object volume, closed porosity (i.e., pores not connected to the surface in the micro-CT images), number of closed pores, and distribution of closed pore sizes were performed with CTAn (Bruker microCT, Kontich, Belgium). DataViewer (Bruker microCT, Kontich, Belgium) was used for visualization of cross sections and measurements of specimen diameters. Pore size distribution in cements was analysed using volume-equivalent sphere diameter and counting the number of pores within different ranges. A lognormal distribution function was found to describe the pore size distribution well and has previously been described in the literature of hardened cement pastes and concrete [23]. The distribution fitting tool (dfittool) in MATLAB (version R2012a, The MathWorks Inc., Natick, MA, USA) was used for the curve fitting.

### 2.5. Mechanical Testing

Cement specimens (12 replicates per liquid and time point, plus 5 replicates per liquid from the micro-CT study) were tested in quasi-static compression using a materials testing machine (AGS-X, Shimadzu, Kyoto, Japan), equipped with a 5 kN load cell, using a displacement rate of 1 mm/min. All specimens were polished plane-parallel when needed (method described above) and were kept wet until testing.

### 2.6. Phase Characterization

XRD (D8 Advance, Bruker, AXS GmbH, Karlsruhe, Germany) was used to analyse the phase composition of the cements. Ni-filtered Cu K*α* irradiation, a beam knife, and a theta-theta setup were used for the acquisition. Diffraction patterns were collected between 2*θ* of 10 and 60 degrees, in steps of 0.02 degrees, using 0.25 seconds per step, with a sample rotation speed of 80 rpm. Cement specimens were, after mechanical testing, ground and homogenized into a single quantity of material, for each liquid. Six specimens were taken at random from this quantity and analysed in XRD. Quantitative phase composition analysis was performed using Profex (http://profex.doebelin.org/) [24] as a graphical user interface for the Rietveld refinement program BGMN (http://www.bgmn.de/) [25, 26]. The reported result was the mean of six independent measurements with the repeatability taken as 2.77 × standard deviation according to ASTM E177-14 [27, 28]. Crystalline models were taken from PDF# 04-008-8714 [29] for *β*-TCP, PDF# 04-013-3344 [30] for brushite, PDF# 04-009-3876 [31] for beta-dicalcium pyrophosphate (*β*-CPP), PDF# 04-009-3755 [32] for monetite, and PDF# 04-013-3883 [33] for OCP. No other phases were identified in the diffraction patterns.

### 2.7. SEM

The microstructure of cross sections of the cements was visualized by SEM (TM-1000, Hitachi, Tokyo, Japan) using a backscattered electron detector and an acceleration voltage of 15 kV, at time points 0, 10, and 25 weeks. The specimens were dried under vacuum for 24 hours before analysis to ensure completely dry specimens. A gold/palladium layer, approximately 5 nm thick, was sputtered onto the surface prior to analysis.

### 2.8. Statistical Analysis

IBM SPSS Statistics (Version 19, IBM Corp., Armonk, NY, USA) was used for the statistical analysis. Analysis of variance (ANOVA) was used to compare properties of the cements incubated in the three different liquids at time points 0 and 25 weeks, at a significance level of *α* = 0.05. Scheffe's* post hoc* test was used to compare change in volume over time. To compare porosities and compressive strengths between groups and over time, Tamhane's* post hoc* test was used since Levene's test did not confirm homogeneity of variances.

### 2.9. Correlation between Quasi-Static Compressive Strength and Porosity

The porosity, as evaluated by gravimetric analysis, was correlated to the quasi-static compressive strength for each cement specimen, by fitting an exponential equation to the data [34]:

(3) $$\begin{array}{c} \sigma_{C}=\sigma_{C0}e^{-q\Phi }, \end{array}$$

where *σ*_*C*_ is the compressive strength, *σ*_*C*0_ is the compressive strength of a fully dense cement (zero porosity), Φ is the porosity, and *q* is a dimensionless constant. The curve fitting toolbox (cftool) in MATLAB (version R2012a, The MathWorks Inc., Natick, MA, USA) was used for the curve fitting.

## 3. Results

### 3.1. Gravimetric Analysis by Solvent Exchange

**Figure 2:**
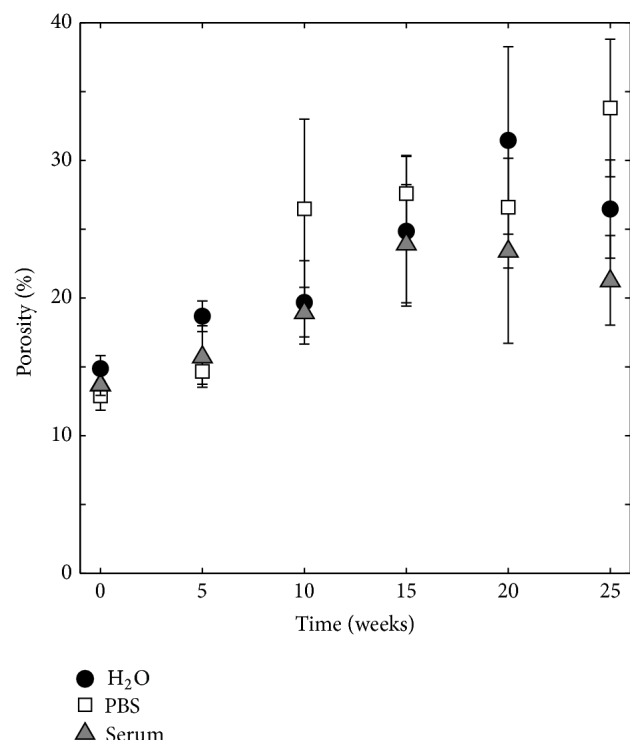
Porosity as a function of degradation time in H_2_O, PBS, and serum solution, *n* = 12.

The brushite cements had an open porosity of approximately 13% after they had set for 24 hours (see Figure 2). Since the cement specimens were prepared in different batches, a small variation in porosity could be seen at time point 0, with no significant differences between H_2_O and serum solution (*p* = 0.059) and between PBS and serum solution (*p* = 0.438), but a significant difference between H_2_O and PBS (*p* ≤ 0.001). Degradation of the cement specimens resulted in a significant increase (*p* ≤ 0.001) in porosity and following 25 weeks of degradation the resulting porosity was 26.5 ± 3.6%, 33.8 ± 5.0%, and 21.3 ± 3.3% for cement specimens soaked in H_2_O, PBS, and serum solution, respectively, with significant differences between all groups (H_2_O/PBS: *p* = 0.008; H_2_O/serum solution: *p* = 0.018; and PBS/serum solution: *p* ≤ 0.001).

**Figure 3:**
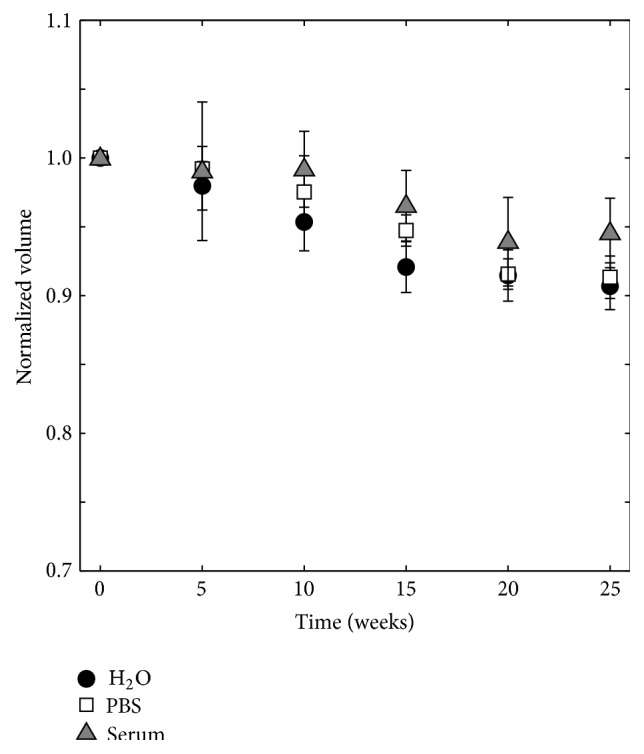
Normalized volume, determined by gravimetric analysis, for specimens kept in H_2_O, PBS, and serum solution, *n* = 12.

The change in specimen volume determined by gravimetric analysis can be seen in Figure 3. A significant decrease (*p* ≤ 0.01) in volume of 9.3 ± 1.7% could be seen when specimens were kept in H_2_O for 25 weeks. For specimens kept in PBS for 25 weeks, the decrease in volume was 8.7 ± 1.5%, and for those kept in serum solution the decrease was 5.4 ± 2.5% (both significant in comparison to time point 0, *p* ≤ 0.01).

### 3.2. Volumetric Analysis by Micro-CT

**Figure 4:**
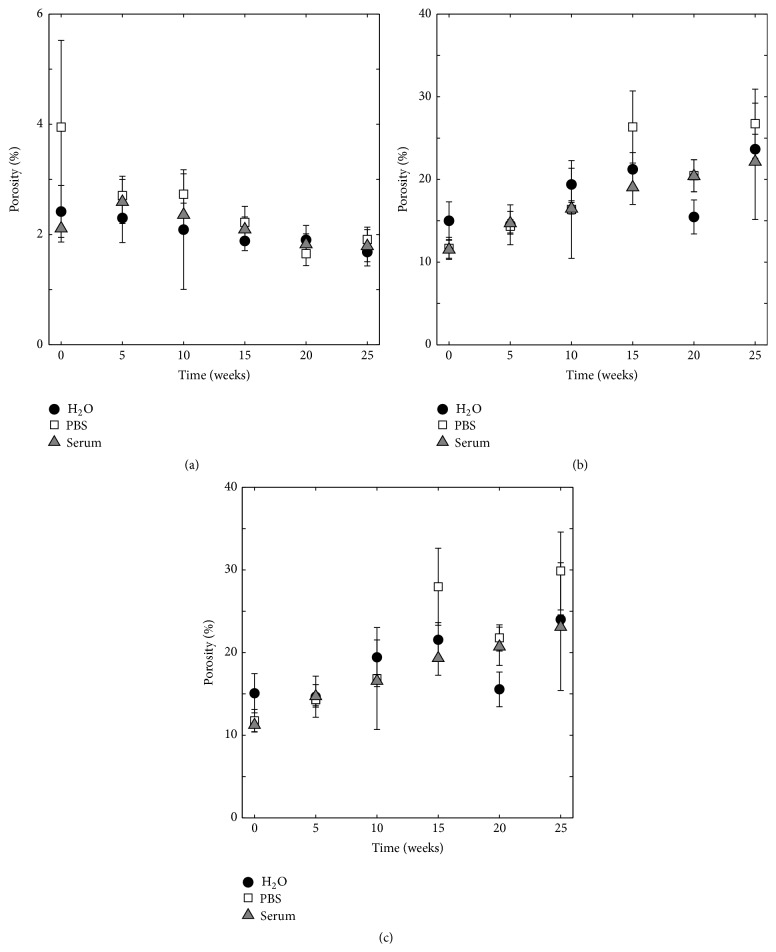
Comparison between (a) closed porosity determined by volumetric analysis, (b) open porosity determined by gravimetric analysis, and (c) open porosity determined by gravimetric/volumetric analysis (*m*_air_ and *m*_solvent_ in (2) taken from gravimetric analysis, *V*_*a*_ from volumetric analysis), *n* = 5.

A comparison between the closed porosity from micro-CT analysis, the open porosity from solvent exchange, and the open porosity determined by the combination of solvent exchange and micro-CT analysis, for the same cement specimens, can be seen in Figure 4. The closed porosity as observed by micro-CT (Figure 4(a)) was similar for specimens kept in H_2_O, PBS, and serum solution and decreased slightly over time (from 2.8% on average to 1.8%). However, the initial, open, porosity obtained from gravimetric analysis (Figure 4(b)) was almost five times higher (approximately 13%), compared to what was observed with the micro-CT. After 25 weeks in H_2_O, PBS, and serum solution, the porosity from gravimetric analysis of these batches was 23.7 ± 1.8%, 26.7 ± 4.2%, and 22.2 ± 7.0%, respectively. The open porosity was also determined from a combination of gravimetric and volumetric analysis (Figure 4(c)), with the apparent volume taken from micro-CT analysis, and the mass of a wet specimen in air and the mass of a specimen after 24 hours in isopropanol taken from gravimetric analysis. This porosity was similar to the open porosity determined solely by gravimetric analysis, and after 25 weeks it was 24.0 ± 0.5%, 29.9 ± 4.7%, and 23.1 ± 7.7 % for specimens incubated in H_2_O, PBS, and serum solution, respectively.

**Figure 5:**
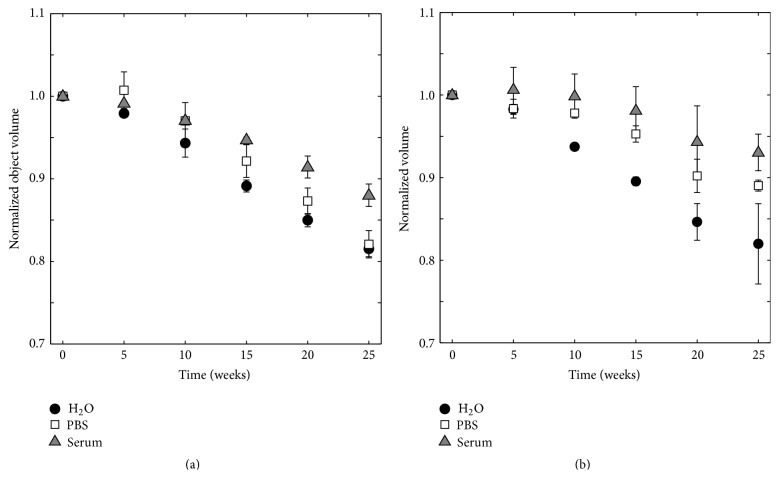
Comparison between (a) normalized object volume from volumetric analysis and (b) normalized volume from gravimetric analysis, for specimens kept in H_2_O, PBS, and serum solution, *n* = 5.

The volume of the specimens from the micro-CT study (5 replicates) was determined in two different ways: by gravimetric analysis and by volumetric analysis (see Figure 1). The object volume, determined by micro-CT, decreased during degradation in all three liquids (Figure 5(a)), which was also the case for the volume as determined by gravimetric analysis (Figure 5(b)). The specimens kept in H_2_O and PBS lost most volume over time, which was a significant decrease (*p* ≤ 0.01), while specimens immersed in serum solution had a larger object volume than the others after 25 weeks of degradation, but still a significant decrease in comparison to time point 0 (*p* ≤ 0.01). At the last time point of the study, the specimens had lost 18.5 ± 1.0%, 17.9 ± 1.7%, and 12.0 ± 1.4% of object volume, as determined by micro-CT (Figure 5(a)), and 18.0 ± 4.9%, 11.0 ± 0.6%, and 7.0 ± 2.2% of volume, as determined by gravimetric analysis (Figure 5(b)), for specimens degraded in H_2_O, PBS, and serum solution, respectively.

**Figure 6:**
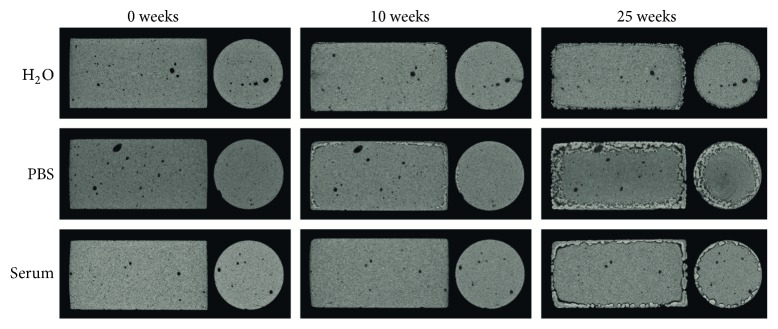
Representative cross sections of cements at time points 0 (left), 10 (middle), and 25 (right) weeks degraded in H_2_O (top), PBS (middle), and serum solution (bottom).

As the cements degraded, visual inspection revealed fragmentation of the surface. From micro-CT cross sections, changes on the outer surfaces of the cement specimens could be seen for all cement samples from week 10 onward. After 25 weeks, a layered structure was more or less pronounced. Representative cement cross sections can be seen in Figure 6.

**Figure 7:**
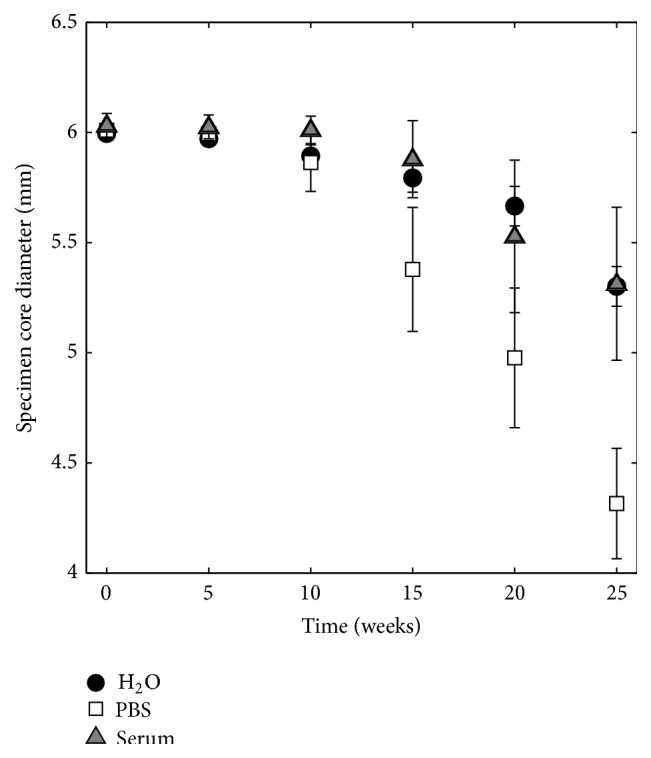
Change of specimen core diameter over time in H_2_O, PBS, and serum solution, *n* = 5.

The diameter of the cement core, that is, the diameter excluding the thin outer layer that was formed, was measured for all specimens at time points 0, 5, 10, 15, 20, and 25 weeks. The core diameter as a function of immersion time can be seen in Figure 7. The specimens aged in H_2_O and serum solution had a similar behaviour, whereas for specimens kept in PBS the layer grew much thicker, together with a concomitantly decreasing core diameter, in accordance with Figure 6.

**Figure 8:**
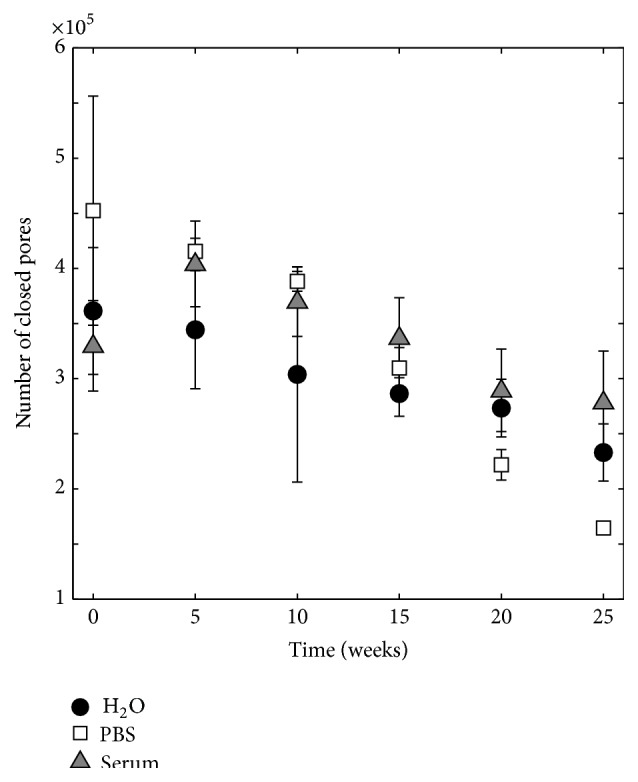
Number of closed pores as a function of time for cements aged in H_2_O, PBS, and serum solution, *n* = 5.

The number of closed pores was counted and analysed over degradation time (see Figure 8). At time point 0, the number of closed pores was 361 447 ± 57 575, 452 445 ± 103 883, and 329 866 ± 41 050 for specimens degraded in H_2_O, PBS, and serum solution, respectively. In general, a decrease in the number of closed pores over degradation time could be seen, and after 25 weeks the number of closed pores was 233 081 ± 25 932, 164 612 ± 2 743, and 278 668 ± 46 396 for specimens degraded in H_2_O, PBS, and serum solution, respectively.

**Figure 9:**
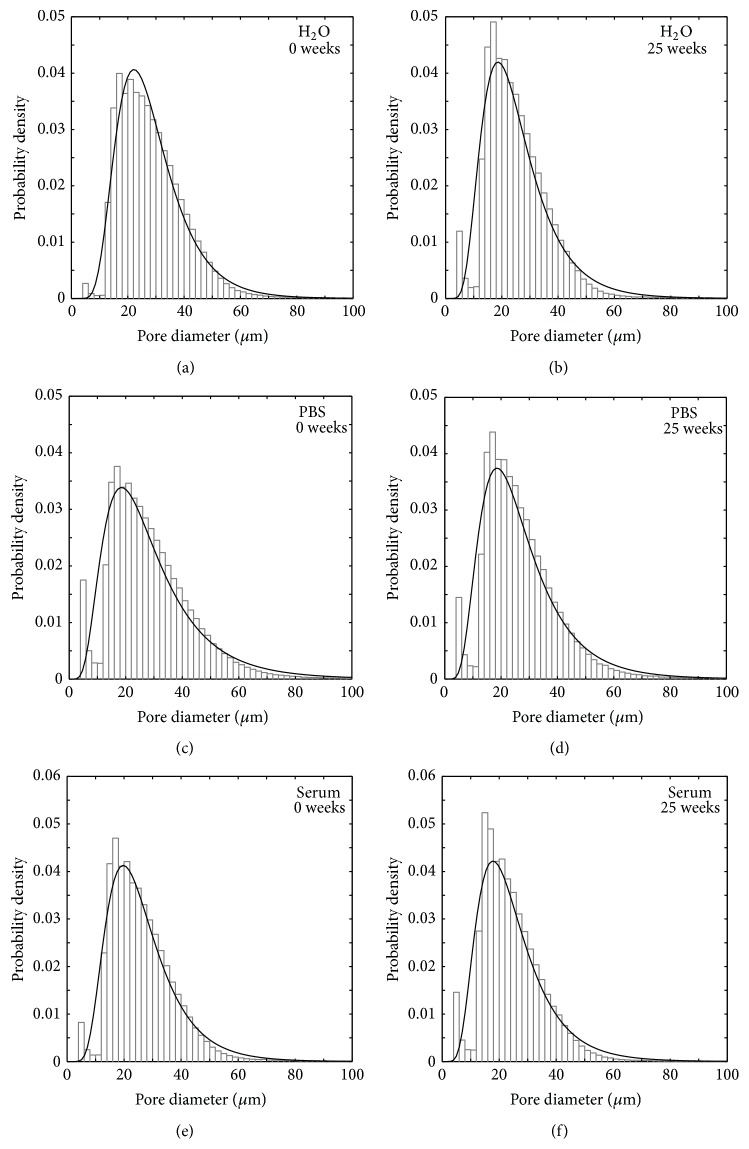
Representative histograms showing pore size distribution in cements degraded in H_2_O ((a) and (b)), in PBS ((c) and (d)), and in serum solution ((e) and (f)). The black lines show a fit to a lognormal distribution. The largest pores had a diameter of about 800 *μ*m and typically 99.8% of the total number of pores had a diameter smaller than 100 *μ*m.

Histograms of pore diameter distributions (closed pores) can be found in Figure 9, together with fits to a lognormal distribution function.

**Figure 10:**
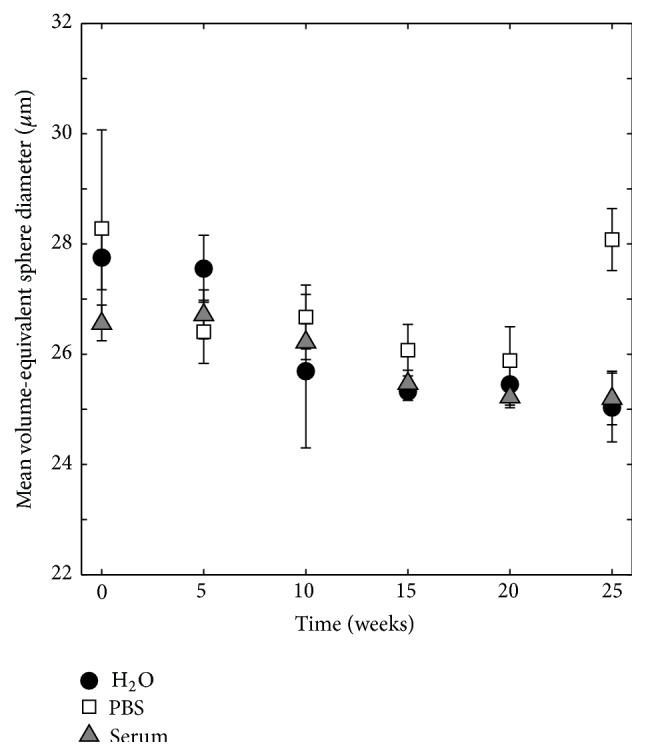
Mean volume-equivalent sphere diameter as a function of time in H_2_O, PBS, and serum solution, *n* = 5.

The mean pore size was calculated from the lognormal data fits for each time point. As can be seen in Figure 10, the mean pore diameter at time point 0 was 27.8 ± 0.6 *μ*m, 28.3 ± 1.8 *μ*m, and 26.6 ± 0.3 *μ*m for specimens degraded in H_2_O, PBS, and serum solution, respectively, and decreased over 25 weeks for all three liquids, except for specimens soaked in PBS which saw a rapid increase in mean pore diameter at time point 25. After 25 weeks, the mean pore size was 25.0 ± 0.6 *μ*m, 28.1 ± 0.6 *μ*m, and 25.2 ± 0.5 *μ*m for specimens degraded in H_2_O, PBS, and serum solution, respectively.

### 3.3. Mechanical Testing

**Figure 11:**
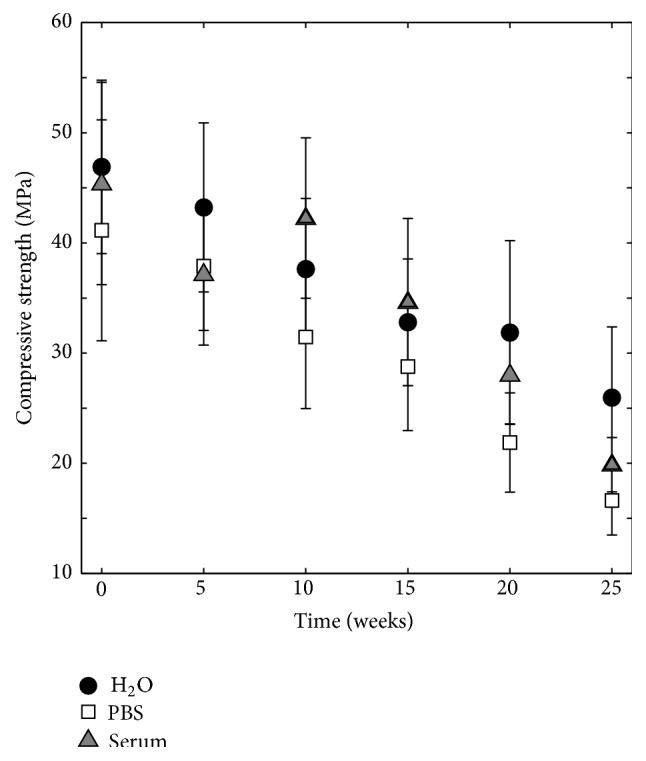
Compressive strength for specimens kept in H_2_O, PBS, and serum solution, *n* = 12.

Quasi-static compressive strengths of wet cements can be seen in Figure 11. Initial mean strengths after 24 hours of setting were between 41.1 and 46.9 MPa. The strength of all specimens decreased significantly (*p* ≤ 0.01) over time. After 25 weeks, the compressive strength was 26.0 ± 6.4 MPa, 16.6 ± 3.1 MPa, and 19.9 ± 2.5 MPa for specimens degraded in H_2_O, PBS, and serum solution, respectively, with no significant differences between H_2_O and serum solution (*p* = 0.118) and between PBS and serum solution (*p* = 0.143) and a significant difference between H_2_O and PBS (*p* = 0.005).

As a control, the quasi-static compressive strength of specimens that had been analysed with micro-CT was tested after 25 weeks of degradation in H_2_O, PBS, and serum solution (see Figure 1), and the strength was 28.6 ± 3.9 MPa, 17.9 ± 1.4 MPa, and 25.9 ± 9.9 MPa for specimens degraded in H_2_O, PBS, and serum solution, respectively, which were not significantly different to the strengths of the specimens in the other series of experiments (Figure 1) (*p* ≥ 0.986).

### 3.4. Phase Characterization

**Figure 12:**
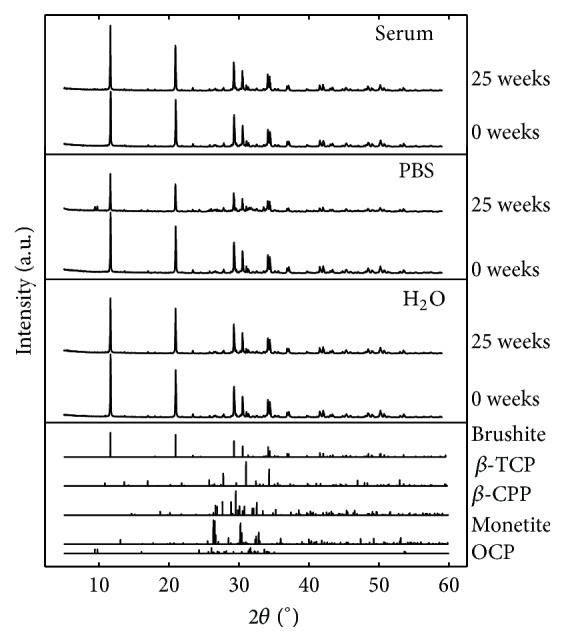
XRD patterns for specimens kept in H_2_O, PBS, and serum solution after 0 and 25 weeks (one out of six measurements for each liquid and time point is shown).

XRD patterns from phase analysis of specimens kept in PBS, H_2_O, and serum solution for 0 and 25 weeks are shown in Figure 12 (one representative pattern for each group and time point), together with reference patterns for the identified phases. The patterns collected after 5, 10, 15, and 20 weeks (for each respective liquid) looked similar to those shown in Figure 12.

**Figure 13:**
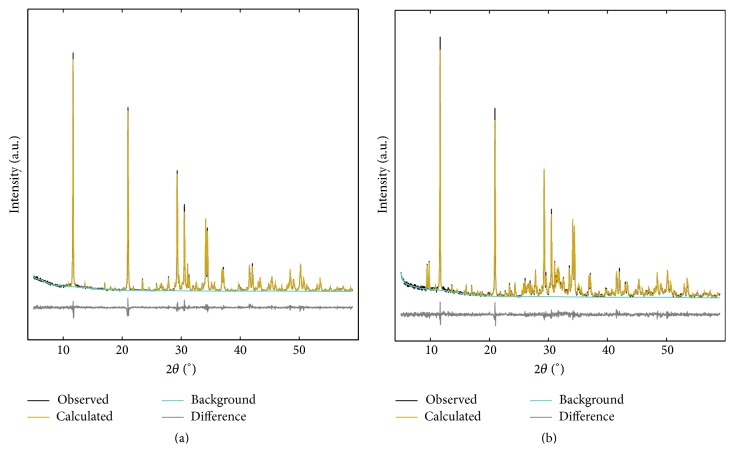
Representative XRD patterns showing the accuracy of the refinement, from time points (a) 0 weeks and (b) 25 weeks.

Figure 13 shows the accuracy of the Rietveld refinement, for patterns collected at time points 0 and 25 weeks. These specific patterns come from the refinement of patterns for specimens kept in PBS, as these where considered representative. However, the OCP peaks were not as pronounced at 25 weeks for specimens kept in H_2_O and serum solution.

**Figure 14:**
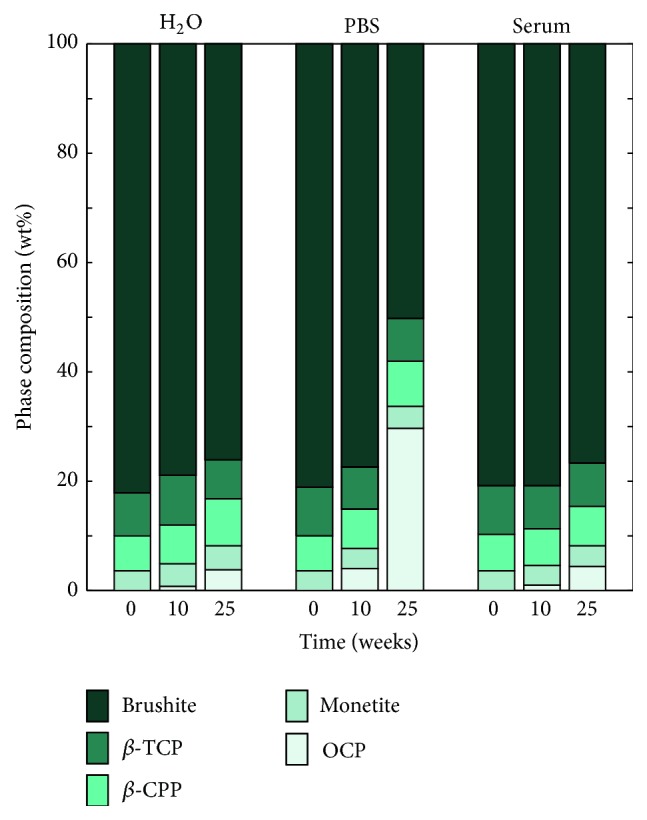
Phase composition of cement specimens after 0, 10, and 25 weeks for specimens kept in H_2_O, PBS, and serum solution, *n* = 6/group. Repeatability was equal to or better than 1.5 wt% for all groups.

Initially, the specimens contained approximately 81 wt% brushite, 8 wt% unreacted *β*-TCP, 7 wt%  *β*-CPP (contamination of the *β*-TCP powder), and 4 wt% monetite (see Figure 14). After 10 weeks, OCP appeared in the patterns for specimens kept in PBS; however, for specimens that were kept in H_2_O and serum solution, only very small amounts (<1 wt%) of OCP could be detected. As can be seen in Figure 14, the specimens kept in PBS contained more OCP after 25 weeks compared to what was found for specimens kept in H_2_O and serum solution.

Phase composition of cement specimens that had been analysed with micro-CT was performed after 25 weeks as a control (see Figure 1), and similar phase compositions as for the other specimens were found for all three liquids (data not shown). After 25 weeks, the outermost layer of the cement specimens was scraped off and analysed separately along with a piece of the core of the specimen, in thin film XRD (same settings as for the powder XRD). These analyses showed that the layer consisted of mostly OCP and that no OCP was present in the core of the specimen. Since the Rietveld calculations were performed on XRD patterns taken when the whole cement specimens, including both the core and the outermost layer of the specimen, were ground and homogenized into a powder, it is likely that the OCP present in all cement specimens (Figure 14) actually came only from the periphery of the specimens. This observation could also explain that the specimens degraded in PBS had a greater amount of OCP compared to H_2_O and serum solution, since those specimens also revealed the thickest peripheral layer (Figures 6 and 7).

### 3.5. SEM

**Figure 15:**
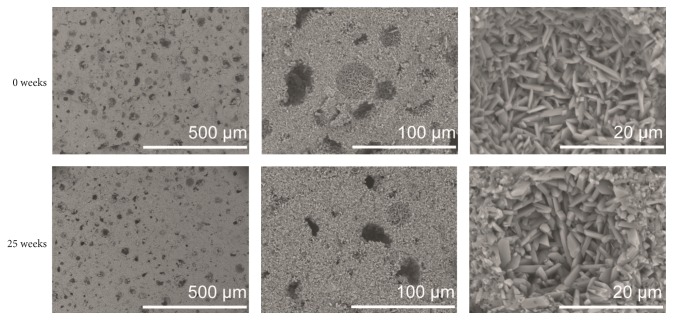
Representative SEM images of cements at time points 0 (top) and 25 (bottom) weeks. Three different magnifications are shown. This particular cement specimen was kept in H_2_O, but all three liquids showed similar microstructures.

Visualization of cements by SEM after 0, 10, and 25 weeks of degradation showed no apparent differences in microstructure between different time points and between the three liquids. Representative images of cements at time points 0 and 25 weeks can be found in Figure 15.

**Figure 16:**
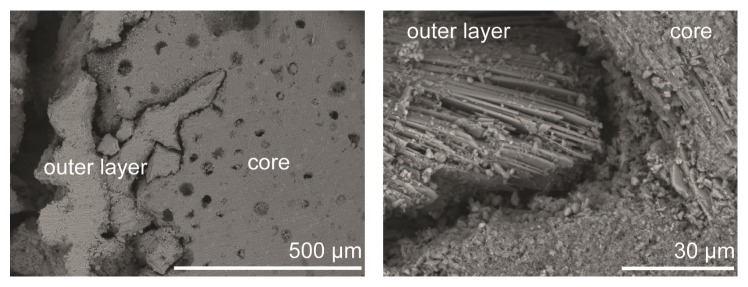
SEM images showing the core and the outermost layer of a cement specimen, at two different magnifications. This specific specimen had been kept in PBS.

SEM images of the outermost layer of the cement specimens revealed a layered structure, as can be seen in Figure 16.

### 3.6. Correlation between Quasi-Static Compressive Strength and Porosity

**Figure 17:**
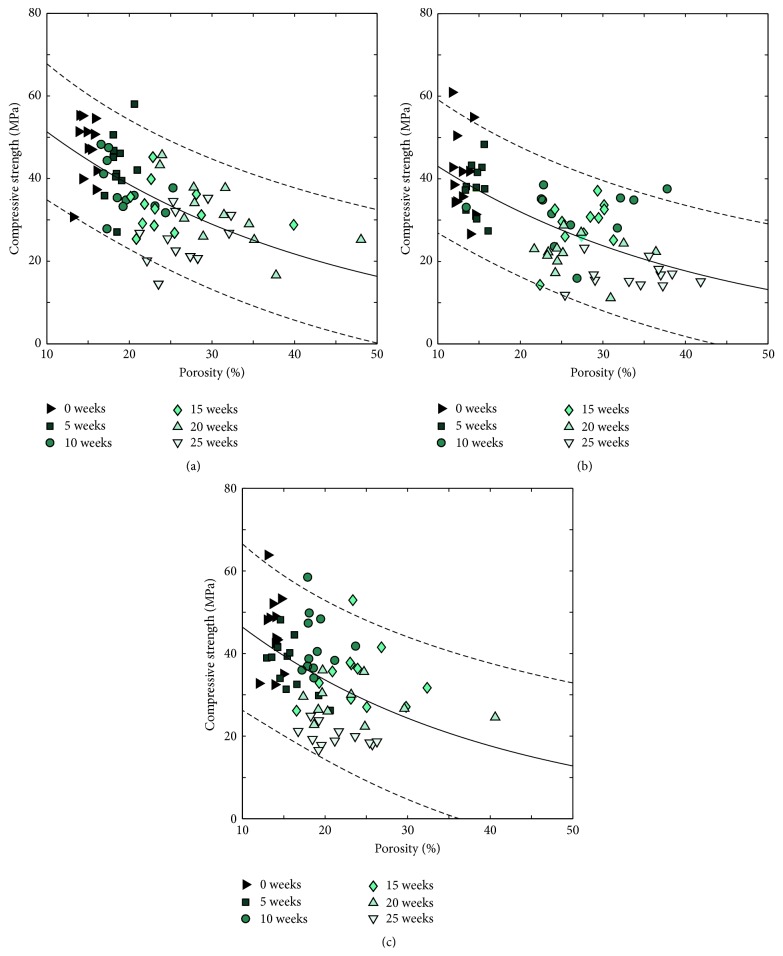
Correlation between compressive strength and porosity as evaluated by gravimetric analysis for specimens kept in (a) H_2_O, (b) PBS, and (c) serum solution (*n* = 12 for all groups). Fits to (3) are shown as a continuous line and 95% confidence intervals are shown as dotted lines.

Figure 17 shows the correlations between quasi-static compressive strength and porosity for specimens degraded in the three different liquids. As expected, a clear trend was seen for all three liquids: the compressive strength decreased with an increase in porosity.

## 4. Discussion

The focus of this study was to evaluate the long-term* in vitro* degradation properties of a high-strength brushite CPC in different solutions. Initially, the cements had similar physicochemical properties to those previously reported for the same cement composition in terms of porosity (13.8 ± 0.9 versus 12.5 ± 1.6%), compressive strength (44.5 ± 9.0 versus 55.1 ± 10.2 MPa), and phase composition (81 wt% brushite, 8 wt%  *β*-TCP, 7 wt%  *β*-CPP, and 4 wt% monetite versus 82 wt% brushite, 8 wt%  *β*-TCP, 6 wt%  *β*-CPP, and 4 wt% monetite) [13]. An increase in porosity with a concomitant decrease in strength over time was seen for all three degradation liquids. The phase composition of the cements was also affected by the degradation.

The increase in porosity, as determined by gravimetric analysis, was on average 0.3–0.8 percentage points per week (Figure 2). This is a lower porosity increase compared to what has previously been reported by Grover et al. [6] (on average 3.8 percentage points per week, as determined by helium pycnometry [6]). Moreover, the decrease in strength over time was on average 0.8–1.0 MPa per week (Figure 11), compared to 1.5 MPa per week [6]. However, these values are not directly comparable as the study of Grover et al. [6] differs in terms of cement composition and consequently physical properties (initial strength, 14 ± 2 MPa, and a higher porosity, 21 ± 1%) and degradation protocol (the study length was shorter, 4 weeks, and the PBS was refreshed on a daily basis) from the study herein. It can be noted that the initial porosity is likely to have a large effect on the degradation rate.

The degradation of the brushite cements reflected the changes that occurred on the cement surface, rather than any changes happening in the core of the cements (Figures 6, 15, and 16). After 10 weeks, the outermost layer of the cements had changed macroscopically for specimens degraded in all three liquids, which was also obvious from micro-CT cross sections (Figure 6) and analysis of specimen core diameter (Figure 7). Specimens in PBS showed greater differences in terms of specimen core diameter and visual appearance of cross sections, as compared to H_2_O and serum solution, which had more similar appearances. Coming closer to the end of the study (20–25 weeks), it was apparent that the cements had degraded through considerable fragmentation. The same degradation mechanism, that is, disintegration of the cements, has been shown before, but for much weaker brushite cements, containing considerable amounts of unreacted *β*-TCP (initially 66 wt%), and kept in a calcium-containing PBS or undiluted FBS over a time period of 4 weeks [5]. Grover et al. found that specimens kept in FBS degraded much faster compared to those kept in PBS (Table 1; the change in mass was 16 percentage points/week in FBS compared to 3 percentage points/week in PBS, on average) due to the formation of a more stable phase (HA) in the latter, thus slowing down the degradation rate. Their results contradict the present findings; that is, the loss in volume (Figures 3 and 5) was herein on average the lowest for specimens kept in serum solution (0.48 percentage points/week in object volume, Figure 5(a), corresponding to approximately 0.22 percentage points/week in mass (calculated from the change in* V*_*a*_ and the density of the cement)) compared to H_2_O (0.74 percentage points/week in object volume, Figure 5(a), corresponding to approximately 0.37 percentage points/week in mass) and PBS (0.72 percentage points/week in object volume, Figure 5(a), corresponding to approximately 0.35 percentage points/week in mass). Grover et al. concluded that FBS decreased the dissolution rate of brushite and inhibited the formation of HA and that the refresh rate and composition of degradation media are critical to the degradation mechanisms seen* in vitro* [5]. An overview of degradation rates found in the literature for brushite cements can be found in Table 1. Besides differences between degradation media, a general trend for higher degradation rate with a higher L/P ratio can be discerned, in accordance with the increase in porosity that is generally found for higher L/P ratios. In fact, our low L/P ratio and hence low initial porosity gave lower degradation rates than previous studies.

The disintegration of the cement specimens seen in this study can also explain the increasing variation in porosity (by gravimetric analysis) that was seen to occur from 10 weeks onwards: pieces of the outermost layer of the cements were falling off during gravimetric analysis, even though the specimens were treated in a very gentle way. Hence, it is important to evaluate not only the porosity change over time, but also the change in volume (or mass) to be able to fully characterize the degradation properties of the cements. The fragmentation of the specimen surface can further explain the high standard deviation found for the volume change, as determined by gravimetric analysis (Figure 3). Comparing SEM images of the specimens, taken after 24 hours of setting and after the cements had degraded for 10 and 25 weeks, it was evident that the microstructure of the cement cores did not change throughout the study (time points 0 and 25 shown in Figure 15) but that the surface layer accounted for most changes in the microstructure (Figure 16). Micro-CT analysis also did not show any major differences in cement core appearance throughout the study (Figure 6).

As expected, the porosity obtained by volumetric analysis was found to differ greatly from the one obtained by gravimetric analysis (Figure 4). While solvent exchange (gravimetric analysis) takes into account all pores that are open and can be penetrated by the isopropanol molecule, it neglects closed pores/pores with entrances smaller than ~9 Å [13]; the micro-CT analysis can only account for pores that have sizes larger than the resolution of the scanner (in this case a voxel size of 6.9 *μ*m^3^). A previous study of the pore size distribution of a similar cement (same composition, though using a higher L/P ratio compared to the present study), using mercury intrusion porosimetry, showed that most pores had a size around 1 *μ*m, but smaller and larger sizes were also present [35]. This emphasizes one of the current major disadvantages of micro-CT analysis for the determination of porosity of CPC specimens; it is highly dependent on the resolution of the scanner, which at present is not high enough to encompass most pores present in this type of specimens. Nevertheless, the micro-CT (volumetric analysis) was considered a good complement in terms of analysis of closed macroporosity.

The number of closed pores was seen to decrease almost linearly over time (Figure 8). Since most changes of cement macro- and microstructure were seen to happen on the surface of the specimens (Figures 6, 15, and 16), the formation of the porous peripheral layer likely consumed closed pores, resulting in a decrease in number of closed pores. Specimens that were incubated in PBS saw the largest decrease in number of closed pores, which is in agreement with this reasoning. When micro-CT calculations were scrutinized, it could be seen that when pores in the layered structure were interconnected and in contact with the deionized water surrounding the specimens during micro-CT analysis, such pores were not interpreted as closed pores. However, as the outer layer grew thicker over time, it is possible that at later time points some of the pores within this layer were not connected to the surface and, hence, were included in the porosity analysis. This could explain the increase in mean pore diameter that was seen at time point 25 for specimens kept in PBS (Figure 10). For the other degradation liquids and time points, the mean pore diameter was seen to decrease almost linearly over time and was rather similar for the three liquids, likely a result of merging of pores in the periphery of the specimens into a few pores with a larger mean diameter, thus resulting in an overall decrease in the mean pore diameter. However, as already mentioned, the overall mean pore size of a similar cement showed that most pores had a size around 1 *μ*m, but smaller and larger sizes were also present [35], and this should not be confused with the macro pore size obtained from volumetric analysis, taking only closed pores having a size larger than the resolution of the micro-CT scanner into account.

The Rietveld analysis revealed that the phase composition of the cements changed over time; the amount of brushite decreased with a concomitant increase in OCP (Figure 14). The presence of OCP was barely noticeable in the cements until after 10 weeks of degradation, further underlining the importance of a long-term study. The formation of OCP was most prominent for the cements degraded in PBS. However, as mentioned above, the OCP was only present in the outermost layer of the specimens and not in the core of the specimens. OCP is often seen as a precursor in the formation of HA [36], which would slow down the degradation rate of the cements due to the chemical stability of HA. In fact, it has previously been shown that the formation of HA retarded the degradation process in terms of mass loss [5]. However, in the present study, the formation of OCP did not seem to affect the rate of increase in open porosity (from gravimetric analysis), decrease in object volume, and decrease in compressive strength. An explanation for this observation could be the difference in degradation protocols between the studies, for example, composition of media and refresh rate, but the initial composition of the cement (e.g., the addition of pyrophosphate ions) is also likely to affect the degradation properties of the cement.

The compressive strength decreased almost linearly over time but was still after 25 weeks higher than 20 MPa, that is, higher than reported trabecular bone strengths [37–39]. This is much higher compared to previous degradation studies of brushite cements that have focused on cements with a rather low strength, most of them with initial strengths lower than 15 MPa (24 hours after setting) [5–7], with only one exception (27.6 ± 3.3 MPa [9]). Furthermore, when the compressive strength was correlated to porosity (as evaluated by gravimetric analysis), it was seen to fit well to (3) for specimens degraded in all three liquids (Figure 17).

In this study, the degradation liquid was seen to affect the properties of the cements while being incubated for 25 weeks. The properties of the cements soaked in PBS were affected in a different way compared to H_2_O and serum solution, for example, in terms of the thickness of the outer layer. H_2_O is the least representative liquid of physiological conditions but is the easiest to employ; however, none of the liquids used in this study can be directly comparable to the chemical and biological environment* in vivo*.

Even though* in vitro* testing is not the same as* in vivo* testing, in terms of, for example, chemical as well as biological environment and fluid flow,* in vitro* testing of CPCs can still be a good starting point to determine when these cements could be used and what their limitations are in terms of physicochemical properties. Another limitation of this study is the limited resolution of the micro-CT scanner, which makes it complicated to directly compare the porosities determined by the volumetric analysis with the one obtained by the gravimetric analysis. Future studies could include micropore size distribution using, for example, mercury intrusion porosimetry, as a complement to the macroporosity obtained by micro-CT analysis, and the total porosity obtained by, for example, solvent exchange.

In this study, it was shown that the* in vitro* degradation properties of brushite cements underwent major changes from 10 weeks onwards, for example, in terms of porosity, formation of an outer layer of the specimens, object volume, phase composition, and compressive strength. Hence, this study shows the importance of long-term evaluation of similar cement compositions in order to be able to predict their appropriate use as bone repair materials.

## 5. Conclusions

Micro-CT, gravimetric, strength, compositional, and microstructural analyses were used to evaluate the degradation of low-porosity, high-strength brushite cements over a time period of 25 weeks in three different liquids: H_2_O, PBS, and a serum solution. The loss in both volume and mass was lowest for the specimens kept in serum solution, compared to those kept in H_2_O and PBS, which had similar mass and volume losses. The increase in porosity over time was lower than in previous findings. By the end of the degradation study, the strength of the cements was still higher than reported trabecular bone strengths. The current study has demonstrated the importance of performing long-term studies when the* in vitro* degradation of cements is studied, as important changes in the physical and chemical properties of the cements were observed after 10 weeks of incubation time. Micro-CT was found to be a useful analysis technique to observe the degradation propagation in 3D. However, due to the limited resolution of the micro-CT, the study also highlighted the need to complement the micro-CT analysis with other porosity measurement methods when evaluating the properties of brushite cements or, alike, under* in vitro* degradation.

## Figures and Tables

**Table 1:** Summary of *in vitro* degradation rates, in terms of average mass loss per week, of brushite cements found in the literature. A range in degradation rate is given when several groups were studied, for example, variations in cement composition or variations in amount of liquid. Only data from degradation experiments performed at 37°C was included.

L/P ratio	Liquid	Time	Degradation rate [mass percentage points/week]	Reference
0.22 mL/g	H_2_O	25 weeks	0.37	Present study
1.33 mL/g	H_2_O	16 days	7.1–8.9	[10]

0.22 mL/g	PBS	25 weeks	0.35	Present study
0.57 mL/g	PBS	90 days	1.5	[6]
0.5 mL/g	PBS	90 days	1.2–1.9	[19]
0.57 mL/g	PBS	28 days	2.1–4.8	[5]
0.8 mL/g	Ringers solution	28 days	2.8	[7]
0.5 mL/g	PBS	21 days	1.8	[15]
1 g/g	PBS	14 days	2.9–11.7	[14]
1 g/g	PBS	14 days	0.5–8.7	[8]

0.22 mL/g	Serum	25 weeks	0.22	Present study
0.57 mL/g	FBS	28 days	16.0	[5]
0.57 mL/g	FBS	90 days	4.4	[6]
